# Comments on: Gene Polymorphisms in *FAS* (Rs3740286 and Rs4064) are Involved in Endometriosis Development in Brazilian Women, but not those in *CASP8* (rs13416436 and rs2037815)

**DOI:** 10.1055/s-0038-1675197

**Published:** 2018-10

**Authors:** Fabio Barra, Lorenzo Ferro Desideri, Giulio Evangelisti, Matteo Tantari, Carolina Scala, Simone Ferrero

**Affiliations:** 1 Academic Unit of Obstetrics and Gynecology, IRCCS Ospedale Policlinico San Martino, Genoa, Italy; 2 Department of Neurosciences, Rehabilitation, Ophthalmology, Genetics, Maternal and Child Health, University of Genoa, Genoa, Italy

We read with great interest the article by Pissetti et al^1^ entitled ‘Gene Polymorphisms in *FAS* (Rs3740286 and Rs4064) Are Involved in Endometriosis Development in Brazilian Women, but not those in *CASP8* (rs13416436 and rs2037815),’ published in your journal. The authors investigated the association between two polymorphisms involved in apoptosis and the risk of developing endometriosis in a case-control study enrolling 45 women. Interestingly, they found a positive association between polymorphisms of the Fas cell surface death receptor gene *FAS* and the confirmed surgical diagnosis of endometriosis.

The authors investigated polymorphisms of the *FAS* gene, similarly to other previous authors.^2^ Overall, one of the alterations appearing in the eutopic and ectopic endometria of women with endometriosis refers to the regulation of apoptosis. In fact, anomalous susceptibility of endometrial tissue to apoptosis may deeply contribute to the development of implants^3^; in particular, it has been reported that expressions of *FAS* and its ligand may be decreased in eutopic and ectopic endometria of women with endometriosis.^4^ In contrast, higher levels of proapoptotic proteins, such as *FAS* ligand, in the peritoneal fluid of women with endometriosis may contribute to an increased apoptosis of immune cells, leading to decreased scavenger activity in the peritoneal cavity and, thus, less immune clearance.^5^

Pissetti et al^1^ should be congratulated for their laboratory findings regarding a controversial and complex topic. In any case, we would be glad to make some considerations on this interesting study. First, it would be of particular interest to know if the presence of these polymorphisms would be different in patients with implants originating from peritoneal nodules, ovarian endometriomas or deep infiltrating endometriosis, three distinguished phenotypes of endometriosis that are known to have probably different pathogeneses.^6^ In relation to this, it has been previously supposed that apoptosis could especially contribute to the survival of endometrial cells regurgitated in the peritoneal cavity and the subsequent development of peritoneal endometriotic implants.^3^

Although the authors correctly reported the distribution of the different endometriosis stages in the patients' population, according to the American Society for Reproductive Medicine (ASRM) classification, it would be interesting to know if there has been a specific correlation between *FAS* genetic polymorphisms and a higher median endometriosis stage or a worse patients' clinical symptomatology.

In conclusion, the role of apoptosis in endometriosis appears to be an attractive topic; in the near future, this pathway may represent a suitable target for treatment of this disease, which needs a chronic therapy balancing clinical efficacy with tolerability.^7^ In support of this view, in a recent narrative review, we found preclinical experiments in vitro and in animal models focalized on drugs acting on apoptosis.^8^ Furthermore, as stated by the authors, the investigation of these polymorphisms as biomarkers to determine predisposition and prognosis for endometriosis appears another fascinating opportunity.

## Notes

Authors' Reply We thank our colleagues for their correspondence commenting our article published in RBGO,^9^ mainly for their valuable comments. The purpose of our manuscript was to investigate the association between *caspase-8* (*CASP8*) (rs13416436 and rs2037815) and *Fas cell surface death receptor* (*FAS*) (rs3740286 and rs4064) polymorphisms and endometriosis in Brazilian women. In fact, endometriosis is categorized into three types: peritoneal superficial endometriosis (SUP), ovarian endometrioma (OMA) and deeply infiltrating endometriosis (DIE).^10^ This classification relates the disease to its pathophysiology and also to the symptoms presented. The information about the three different types of manifestations is not present in the medical records. Therefore, it was not possible to evaluate the association with the studied polymorphisms. We emphasize the rigorous diagnosis used to include cases and controls in our sample. The presence of endometriosis was verified by laparoscopy or laparotomy in both case and control groups. A recent study evaluated the histological and immunohistochemical characterization of the similarity and difference between OMAs and DIE.^11^ The many similarities shared indicate that the two conditions may actually share the same pathogenesis/pathophysiology. Their differences, however, suggest that the source of these differences may result from the different lesional microenvironments.^11^ The authors suggest that the establishment of a unified histological classification/staging of endometriosis may be within reach. These results reinforce that current clinical practice lacks an adequate definition for endometriosis.^12^
According to the American Society for Reproductive Medicine, endometriosis exists in four stages: I (minimal disease), stage II (mild disease), stage III (moderate disease) and stage IV (severe disease).^13^ This surgical classification is based on the extent of adhesions and size of the lesions. Comparison between rs3740286 and rs4064 polymorphisms genotypes in the *FAS* gene and the different stages of endometriosis (I + II *vs* III + IV) revealed no statistically significant difference (Chi-square test; *p* = 0.15 and *p* = 0.72, respectively), indicating that these polymorphisms are related to the development of endometriosis independent of the disease stage. Although it is not possible to associate the different phenotypes of endometriosis with the studied polymorphisms, our results highlight the importance of apoptosis genes for susceptibility to endometriosis. Still, they open the way to new studies, which may contribute to elucidate the genesis of endometriosis.
